# *Papio* Cranium from the Hominin-Bearing Site of Malapa: Implications for the Evolution of Modern Baboon Cranial Morphology and South African Plio-Pleistocene Biochronology

**DOI:** 10.1371/journal.pone.0133361

**Published:** 2015-08-19

**Authors:** Christopher C. Gilbert, Christine M. Steininger, Job M. Kibii, Lee R. Berger

**Affiliations:** 1 Department of Anthropology, Hunter College of the City University of New York, 695 Park Avenue, New York, NY, 10065, United States of America; 2 PhD Programs in Anthropology and Biology, Graduate Center of the City University of New York, 365 Fifth Avenue, New York, NY, 10016, United States of America; 3 New York Consortium in Evolutionary Primatology, New York, NY, United States of America; 4 Evolutionary Studies Institute, University of the Witwatersrand, Private Bag 3, Wits 2050, Johannesburg, Republic of South Africa; University of Kansas, UNITED STATES

## Abstract

A new partial cranium (UW 88-886) of the Plio-Pleistocene baboon *Papio angusticeps* from Malapa is identified, described and discussed. UW 88-886 represents the only non-hominin primate yet recovered from Malapa and is important both in the context of baboon evolution as well as South African hominin site biochronology. The new specimen may represent the first appearance of modern baboon anatomy and coincides almost perfectly with molecular divergence date estimates for the origin of the modern *P*. *hamadryas* radiation. The fact that the Malapa specimen is dated between ~2.026–2.36 million years ago (Ma) also has implications for the biochronology of other South African Plio-Pleistocene sites where *P*. *angusticeps* is found.

## Introduction

The recent discovery of fossiliferous deposits at Malapa, South Africa, has had a major impact on the study of human evolution [1–3]. Over the past few years, three *Australopithecus sediba* partial skeletons have been described [1, 4–13], and the preparation of additional specimens (including other partial skeletons) is currently underway. Along with the hominin material, numerous faunal specimens from Malapa have also been recovered [2, 14–15], and most are still awaiting formal analysis and description. The description and analysis of the fauna are crucial to understanding aspects of paleoecology, biochronology, and mammalian evolution during the African Plio-Pleistocene. In this paper, we describe the only known non-hominin primate specimen from Malapa, UW 88–886, a specimen of the fossil baboon *Papio angusticeps*. Furthermore, we evaluate *Papio angusticeps* in the broader context of modern baboon craniodental evolution and South African Plio-Pleistocene biochronology.

### Geological context

**Fig 1:**
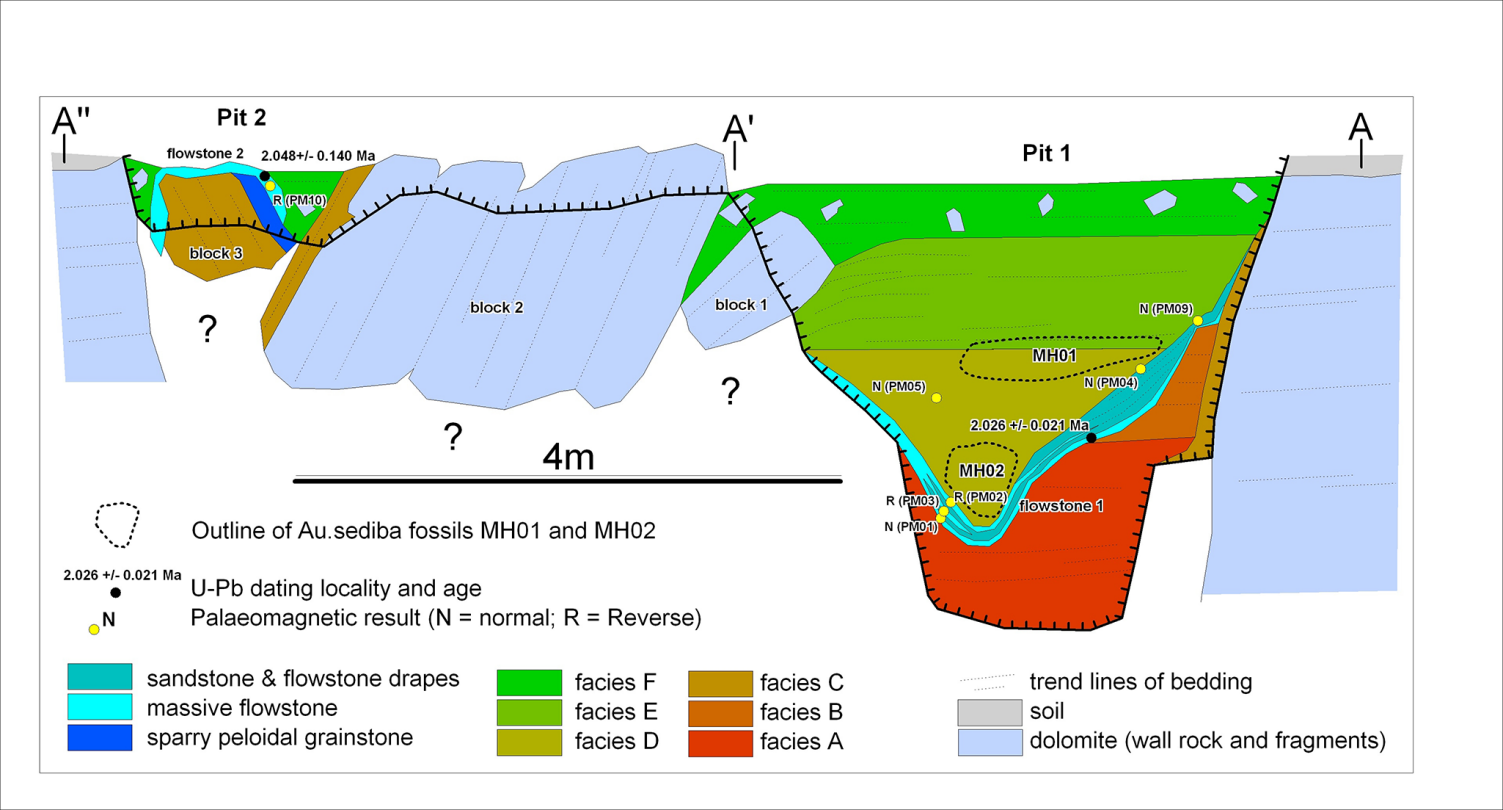
Cross-section sketch map through Malapa showing facies A-F and the U-Pb samples. Modified from [3].

Malapa cave is hosted in the stromatolite-rich, chert-free dolomite of the Lyttelton Formation of the late Archaean (2.64–2.5 Ga) Malmani Subgroup [2], and presently lies near the base of a cave system that was originally >30-m deep prior to erosion by valley incision. Minimal limestone mining in the early 20th century exposed *in situ* cave deposits [2]. Two shallow pits have been recognized at Malapa, of which the main (Pit 1) contains the well- preserved and well-dated (~1.977 Ma) *Au*. *sediba* fossil remains [1–2]. The cave deposits comprise six distinct stratigraphic lithofacies assigned, A to F, from base to top [3]. Facies A and B represent the oldest deposits that were partially eroded before they were covered by flowstone (Flowstone 1, Fig 1). Facies D overlies Flowstone 1, followed by Facies E and topped by Facies F, while Facies C occurs as a remnant between the dolomite on the western sidewall of Pit 1 and Facies B and E (and possibly D) [3]. Flowstone 1 is dated to 2.026 ± 0.021 Ma, suggesting that Facies A and B are older than 2 million years [2]. The *P*. *angusticeps* partial cranium was found in a block of calcified clastic sediments within two metres of Pit 1 and was almost certainly moved to this position by early mining activities. The cranium is embedded within fine-grained clastic dark brown sediment with clastic sandstone. It is unlikely that the specimen comes from Facies A, D and E for a couple of reasons. First, Facies A and D are comprised of course grain sediments, both abundant in fossil remains, and second, Facies E sediments are best characterized as coarse to fine upwards and also contain abundant fossil remains. It is also unlikely that the specimen derives from Facies C and F, since Facies C lacks clastic intercalations while Facies F is devoid of sandstone intercalations. We also considered the possibility that the specimen came from a nearby cave. Indeed, there are two caves in close proximity to Malapa, both less than 150 metres south of the Malapa pits. Currently there are no indications of fossil remains *in situ* or in the mining dumps near these caves.

Therefore, it is most parsimonious to presume that the block containing *P*. *angusticeps* was removed from Pit 1, from Facies B, and the resulting age of this specimen is thus > 2.026 ± 0.021 Ma based on the U-Pb dates of the overlying Flowstone 1 (Fig 1). Geological studies indicate that the different Facies were deposited in quick succession of each other with limited transport distance [2], and the discovery of *Equus* sp., which has its earliest recorded appearance in Africa at ~2.36 Ma [16–18], in Facies D, provides a probable maximum age for the site more generally. Therefore, the probable maximum age for this specimen is ~2.36 million years ago (Ma) based on the appearance of *Equus* sp. in Facies D [2].

## Material and Methods

UW 88–886 is housed in the Evolutionary Studies Institute at the University of Witwatersrand in Johannesburg, Republic of South Africa. Where available, observations and measurements were taken on original specimens using digital calipers and recorded to the nearest tenth of a millimeter. Additional comparative observations and measurements were taken from high-quality casts, the online PRIMO database (access courtesy of Eric Delson), as well as from data kindly provided by Mark Collard to CCG (see Supporting Information, Table A in S1 Dataset). To test for statistical significance between various *Papio* populations, one-way ANOVAs with post-hoc comparisons were computed. Comparative specimens were examined by the authors at the University of Witwatersrand Anatomy Department, Johannesburg (UW-AD), the Bernard Price Institute, Johannesburg (BPI), the Evolutionary Studies Institute, Johannesburg (ESI), the Ditsong National Museum of Natural History, Pretoria (formerly the Transvaal Museum, TM), the Iziko South African Museum, Cape Town (SAM), the University of California Museum of Paleontology, Berkeley (UCMP), and the American Museum of Natural History (AMNH), New York. Bernhard Zipfel, Bonita de Klerk, Steph Potze, Graham Avery and Kerwin von Willingh, Pat Holroyd and Leslea Hlusko, and Eric Delson and Eileen Westwig kindly provided access to primate specimens at UW-AD, BPI/ESI, TM, SAM, UCMP, and AMNH, respectively. For raw measurements used in this study, please see the Supporting Information (Table A in S1 Dataset). All necessary permits and permissions were obtained for the described study, which complied with all relevant regulations. We thank the Nash family and the Malapa Nature Reserve for permission to access the site. We thank the South African Heritage Resource Agency for permission to excavate, Permit No. 80/08/09/001/51 to LRB and JMK.

### Systematic paleontology

Order Primates Linnaeus, 1758

Suborder Anthropoidea Mivart, 1864

Infraorder Catarrhini E. Geoffroy, 1812

Superfamily Cercopithecoidea Gray, 1821

Family Cercopithecidae Gray, 1821

Subfamily Cercopithecinae Gray, 1821

Tribe Papionini Burnett, 1828

Genus *Papio* Erxleben, 1777

*Papio angusticeps* (Broom, 1940)

#### Preservation

The specimen is sheared nearly in half along an approximate sagittal plane thus preserving most of the right part of the cranium. There is little postdepositional compaction with minor broken areas in the maxillary and infratemporal fossae. The preserved portion of the cranium is approximately half-filled with fine sediment capped by a thin layer of flowstone; stage 1 weathering is apparent [19].

#### Description

**Fig 2:**
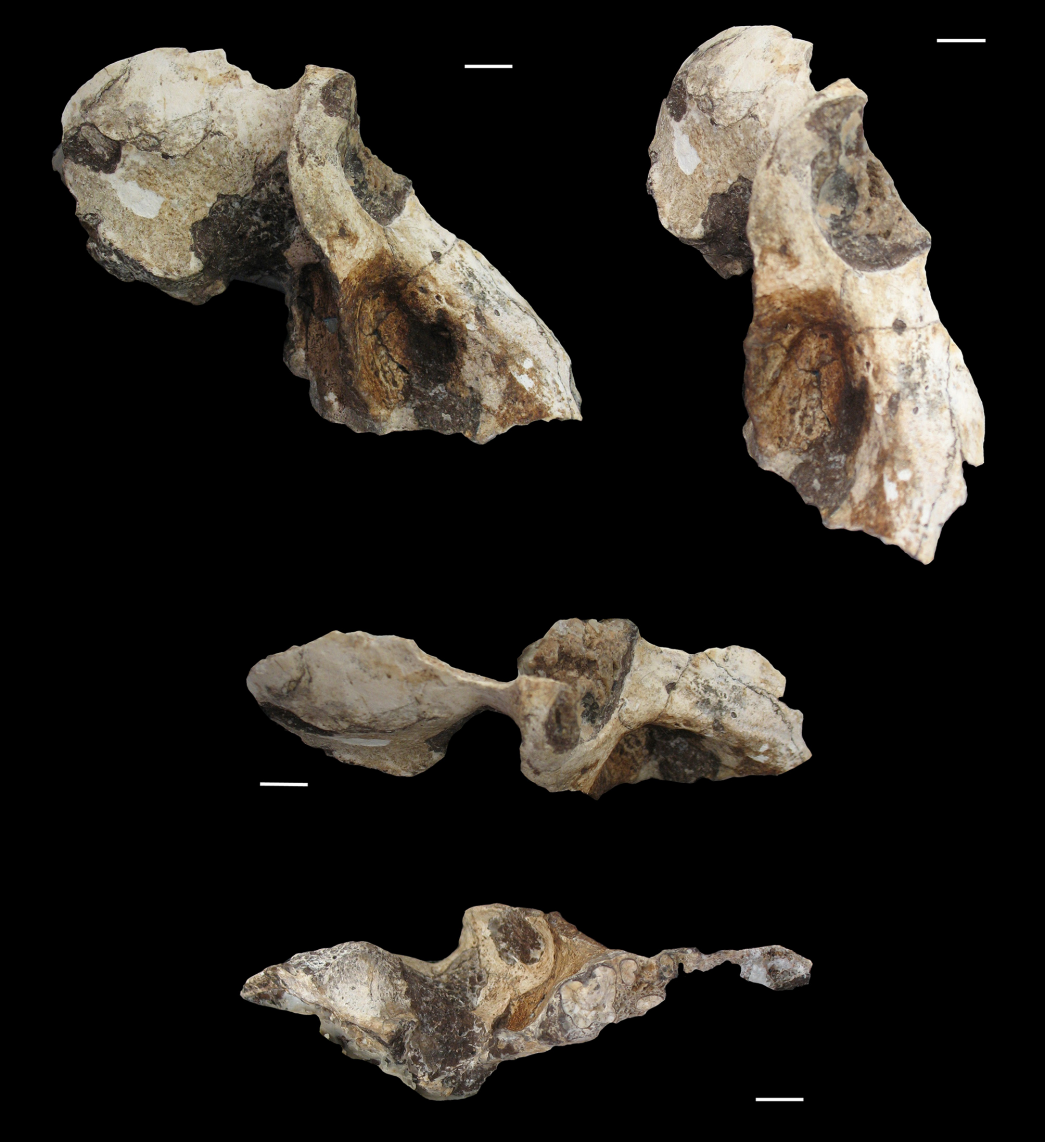
Photographs of UW 88–886, male *P*. *angusticeps* in lateral (top left), oblique (top right), dorsal (middle), and inferior (bottom) views. Note the strong maxillary ridges, deep maxillary fossae, strong temporal lines, and tall malar region, distinctive of *P*. *angusticeps* males. Scale bar in each panel = 1 cm.

**Fig 3:**
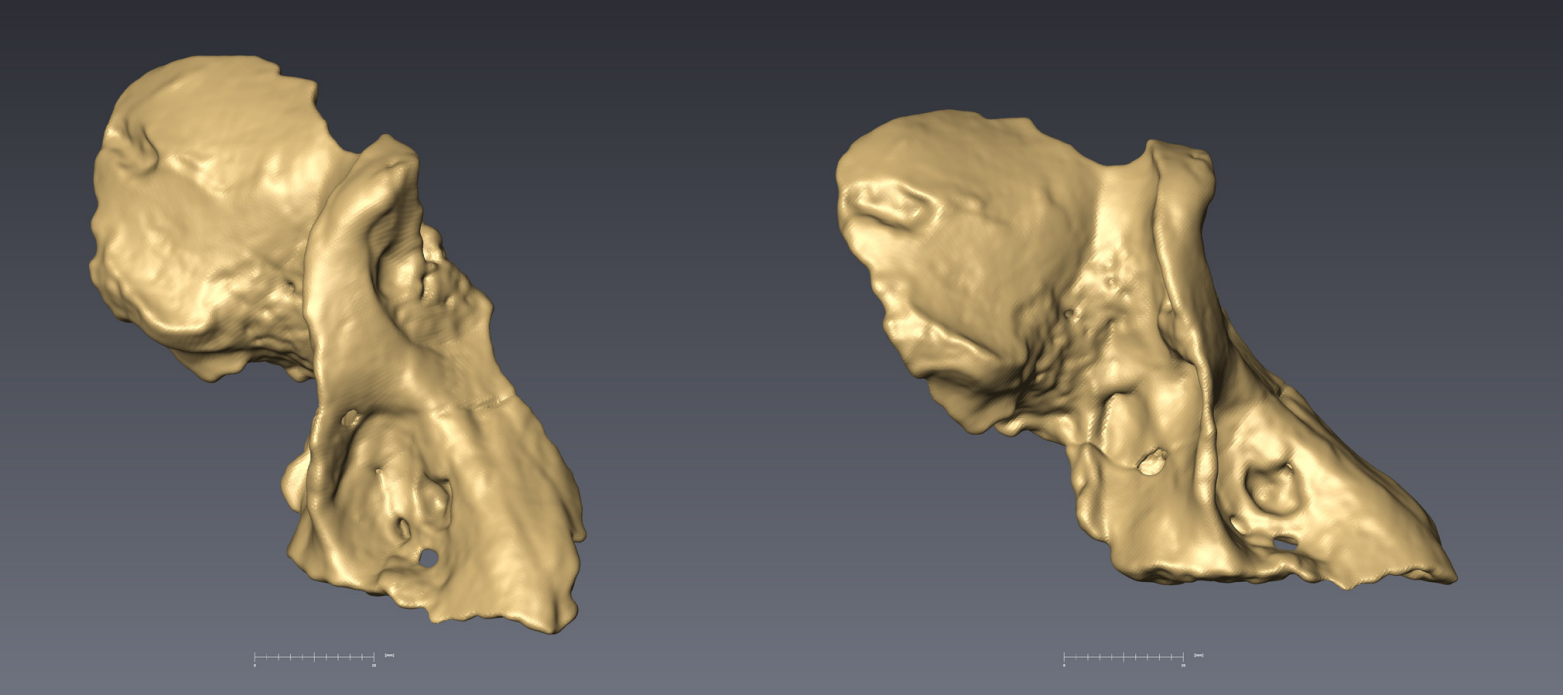
CT Scans of UW 88–866 in oblique (left) and lateral (right) views. Again, note the strong maxillary ridges, deep maxillary fossae, strong temporal lines, and tall malar region, distinctive of *P*. *angusticeps* males.

U.W. 88–886 is a partial cranium preserving most of the right facial skeleton and the right half of the neurocranium (Figs 2 and 3). The overall size, strong maxillary ridges, and strong temporalis muscle markings suggest the specimen is most likely a male. In addition, the overall size of the specimen, apparent anteorbital drop, long and narrow muzzle, strong maxillary ridges, deep maxillary fossae, and tall malar region make the specimen referable to *Papio* among Plio-Pleistocene cercopithecoid taxa, generally, and *P*. *angusticeps* among Plio-Pleistocene *Papio* taxa, more specifically (Table 1). Unfortunately, the dentition is not preserved, with the exception of remnants of the M^2^ and M^3^ roots. The facial skeleton includes portions of the right orbit, right zygomatic, right nasal, and right half of the muzzle including most of the maxilla and a small remnant of the premaxilla (Figs 2 and 3). In addition to the right side of the face, fragments of right frontal, parietal, temporal and zygomatic arch are also preserved. The occipital, basicranium, most of the dorsal surface of the neurocranium, and left side of the cranium have not been recovered.

**Table 1:** Summary of features found in UW 88–886 compared with modern and Plio-Pleistocene *Papio* taxa.

	UW-88-886	*P*. *angusticeps*	*P*. *izodi*	*P*. *robinsoni*	*P*. *hamadryas* ssp.
**Maxillary fossae development**	Well-developed, invade infraorbital plate	Well-developed, invade infraorbital plate	Absent to weakly developed in males, weakly to well-developed and extending up to the infraorbital plate in females	Slightly to well-developed, extend up to infraorbital plate	Well-developed, extend up to and sometimes invade infraorbital plate
**Maxillary ridge development in males**	Present	Present	Absent to weakly present	Present	Present
**Mandibular corpus fossa development**	X	Present and well-developed in males, variably present in females	Absent	Present, slightly developed	Present and well-developed in males, variably present to present and well-developed in females
**Relative malar height**	Tall	Tall	Short	Tall	Tall
**Anteorbital Drop**	Present?	Present	Variably present	Present	Present
**Muzzle shape**	Long and narrow	Long and narrow	Short and broad	Long and narrow	Long and narrow
**Relative tooth size**	X	Small	Large	Small	Small
**Relative orbit size**	Intermediate	Small	Large	Small	Small
**Estimated body mass**	~21 kg?	~21 kg (Males), ~15 kg (Females)	~20 kg (Males) ~15 kg (Females)	~29 kg (Males) ~18 kg (Females)	~16–32 kg (Males) ~10–16 kg (Females)

**Notes:** See text and figures for additional details. Body mass estimates from [39].

**Fig 4:**
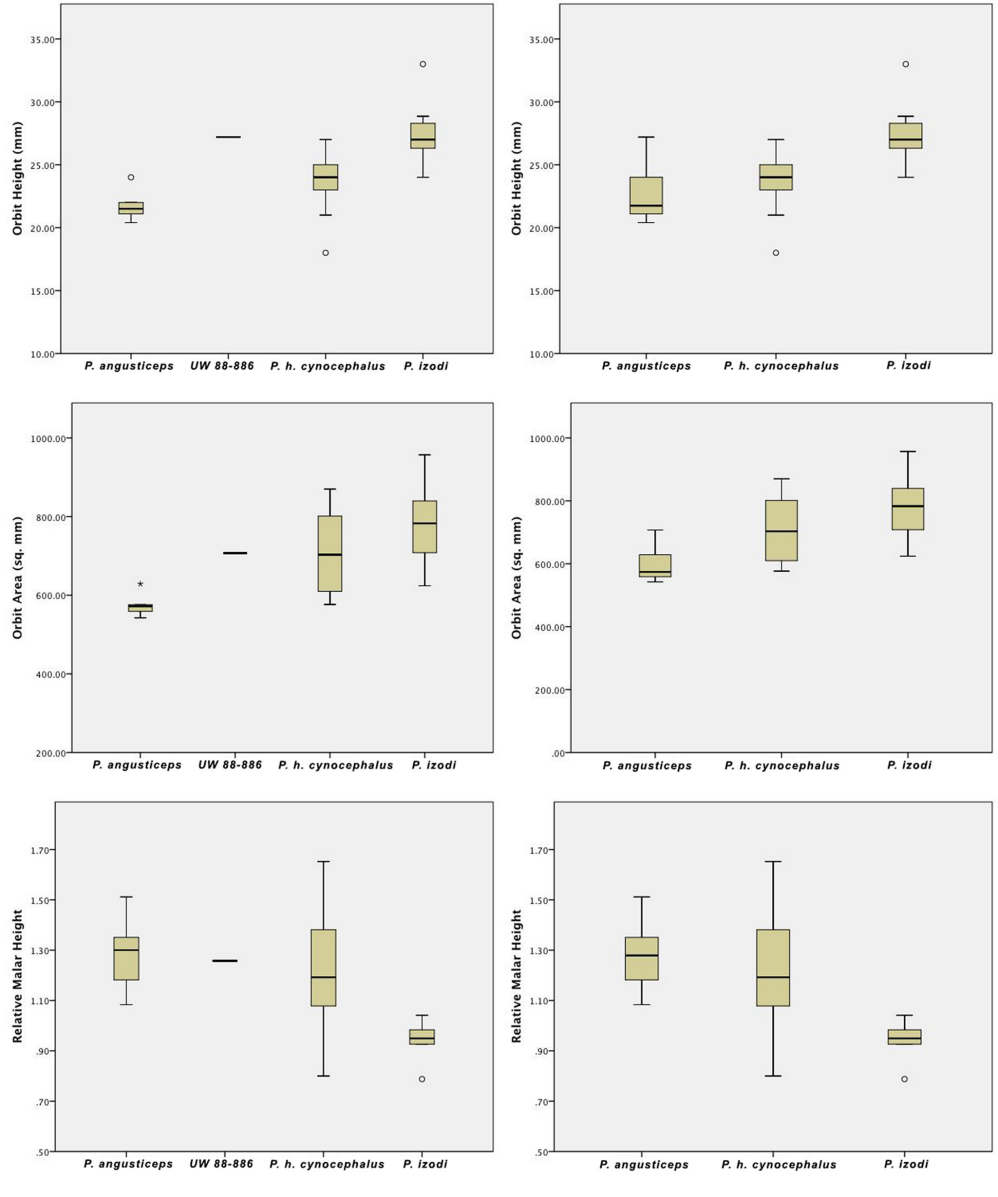
Available craniometric comparisons in *P*. *angusticeps*, UW 88–886, *P*. *h*. *cynocephalus*, and *P*. *izodi*. Top Row: Boxplots of orbit height considering UW 88–886 separately (left) and within *P*. *angusticeps* (right). Note that *P*. *izodi* has significantly taller orbits than both *P*. *angusticeps* and *P*. *h*. *cynocephalus*. UW 88–886 has tall orbits compared to other *P*. *angusticeps* specimens, but within a reasonable range of expected variation for a species. Middle Row: Boxplots of orbit area (mm^2^) considering UW 88–886 separately (left) and within *P*. *angusticeps* (right). *P*. *izodi* has significantly larger orbits than *P*. *angusticeps*, but a non-significant difference compared to *P*. *h*. *cynocephalus*. The difference between *P*. *angusticeps* and *P*. *h*. *cynocephalus* is also non-significant. UW 88–886 appears to have large orbits compared to other *P*. *angusticeps* specimens, but again within a reasonable range of expected variation for a species. Bottow Row: Boxplots of relative malar height considering UW 88–886 separately (left) and within *P*. *angusticeps* (right). *P*. *izodi* has a significantly shorter malar height compared to *P*. *angusticeps* and *P*. *h*. *cynocephalus*, but the difference between *P*. *angusticeps* and *P*. *h*. *cynocephalus* is non-significant. UW 88–886 is closest to the average of other *P*. *angusticeps* specimens. See also Table 2 and Fig 5.

The upper part of the face is distinguished by tall, oval-shaped orbits. Because only half of the orbital rim is preserved (the inferior, lateral, and superolateral aspects), it is impossible to measure the exact size of the orbit, but the orbital height and width can be estimated (Tables 2 and 3; Figs 2–4). Thus, UW 88–886 appears to have large orbits compared to other specimens of *P*. *angusticeps* (a feature more typical of *Papio izodi* among Plio-Pleistocene *Papio* taxa), but additional specimens preserving the entire orbit are needed to confirm this morphology as typical of the Malapa population. Given the other features present in the specimen (see below) and the low sample size of known *P*. *angusticeps* orbits, it seems most reasonable to accept that UW 88–886 simply adds to the known variation of *P*. *angusticeps* at this time. In fact, including UW 88–886 in the existing *P*. *angusticeps* sample of orbit height measurements results in a range of variation (20–27 mm) similar to that seen in a small sample of extant *P*. *h*. *cynocephalus* (18–27 mm, variances statistically equivalent, Levene statistic *p* = 0.344; see Tables 2 and 3; Fig 4). From the preserved anatomy of the upper face and orbital region, particularly the nasals and maxilla just medial to the infraorbital border, it appears that a pronounced anteorbital drop would have been present.

**Table 2:** Comparison of selected morphological features in "small-bodied" *Papio* species.

Taxon	Feature	*n*	Mean	Range	Significance
** **	** **	** **	** **	** **	** **
***Papio izodi***	Orbit Height	10	27.4	24–33	*> P*. *angusticeps*, p < 0.01 > *P*. *h*. *cynocephalus*, p < 0.01
***P*. *angusticeps***	Orbit Height	5	21.8	20–24	< *P*. *izodi*, p < 0.01 *P*. *h*. *cynocephalus*, n.s.
***P*. *h*. *cynocephalus***	Orbit Height	21	23.8	18–27	< *P*. *izodi*, p < 0.01 *P*. *angusticeps*, n.s.
***UW 88–886***	Orbit Height	1	(27.2)	-	-
***Papio izodi***	Orbit Area	8	780	624–957	*> P*. *angusticeps*, p < 0.01 *P*. *h*. *cynocephalus*, *n*.*s*.
***P*. *angusticeps***	Orbit Area	5	576	542–629	*< P*. *izodi*, p < 0.01 *P*. *h*. *cynocephalus*, *n*.*s*.
***P*. *h*. *cynocephalus***	Orbit Area	6	711	577–870	*P*. *izodi*, n.s. *P*. *angusticeps*, n.s.
***UW 88–886***	Orbit Area	1	(707)	-	-
***Papio izodi***	Relative Malar Height	7	0.94	0.79–1.04	*< P*. *angusticeps*, p = 0.02 < *P*. *h*. *cynocephalus*, p < 0.01
***P*. *angusticeps***	Relative Malar Height	5	1.29	1.08–1.51	> *P*. *izodi*, p = 0.02 *P*. *h*. *cynocephalus*, n.s.
***P*. *h*. *cynocephalus***	Relative Malar Height	21	1.22	0.80–1.65	> *P*. *izodi*, p < 0.01 *P*. *angusticeps*, n.s.
***UW 88–886***	Relative Malar Height	1	(1.26)	-	-

**Notes:** Results from one-way ANOVA with Tukey's Honestly Significant Difference post-hoc comparisons for those variables with equal variances and Games-Howell post-hoc comparisons for those variables with unequal variances. Because orbit height, orbit area, and malar height all scale allometrically, the most meaningful comparisons are among taxa of similar body size. The estimated mass for *P*. *angusticeps* averages ~21 kg for males and ~15 for females [39]. *P*. *izodi* is estimated at ~20 kg for males and ~15 for females [39]. The most similar extant taxon in terms of body mass is *P*. *h*. *cynocephalus*, ~23 kg for males and 12.5 kg for females, which is why *P*. *h*. *cynocephalus* is used in the above comparisons. All specimens were pooled regardless of sex in order to increase sample size. For sex-specific values, see Table 3. n.s. = non-significant. Note that *P*. *angusticeps* and *P*. *h*. *cynocephalus* are both significantly different from *P*. *izodi*, but not from each other. Results for all comparisons are the same if UW 88–886 is included in the *P*. *angusticeps* sample. Orbit height defined as the maximum distance between the inferior and superior orbit borders. Orbit width defined as the maximum distance between the lateral and medial orbit borders. Orbit area is defined as orbit width x orbit height. Malar height defined as the distance between orbitale inferior/zygoorbitale and zygomaxillare inferior. Relative malar height defined as malar height/orbit height. *P*. *angusticeps* specimens include CO 100, CO 135A/B, CO 101, GV 4040, and HGD 1249. *P*. *izodi* specimens include TP 12, SAM 11728, T10, T13, UCMP 125854, UCMP 125855, UCMP 125856, STS 262, T89-11-1, and SWP UN-2. Values for each taxon represent averages. Numbers in parentheses represent estimates. For boxplots with ranges, see also Fig 4 and Table A in S1 Dataset.

**Table 3:** Available measurements (in mm) of UW 88–886 Compared with modern and Plio-Pleistocene *Papio* taxa.

	UW 88–886	*P*. *angusticeps* males	*P*. *angusticeps* females	*P*. *izodi* males	*P*. *izodi* females	*P*. *robinsoni* females	*P*. *h*. *cynocephalus* males	*P*. *h*. *cynocephalus* females	*P*. *h*. *kindae* males	*P*. *h*. *kindae* females	*P*. *h*. *anubis* males	*P*. *h*. *anubis* females	*P*. *h*. *ursinus* males	*P*. *h*. *ursinus* females
**Orbit height**	(27.2)	21.3 (*n* = 2)	22.1 (*n* = 3)	28.7 (*n* = 4)	26.2 (*n* = 5)	25.8 (*n* = 3)	24.5 (*n* = 14)	22.8 (*n* = 9)	24.7 (*n* = 8)	23.0 (*n* = 7)	25.6 (*n* = 11)	25.3 (*n* = 12)	25.6 (*n* = 6)	24.6 (*n* = 3)
**Orbit width**	(26)	27.9 (*n* = 2)	25.5 (*n* = 3)	29.5 (*n* = 2)	27.7 (*n* = 5)	29.6 (*n* = 3)	30.7 (*n* = 3)	27.6 (*n* = 3)	29.1 (*n* = 8)	26.7 (*n* = 7)	31.0 (*n* = 7)	29.0 (*n* = 4)	33.1 (*n* = 6)	28.6 (*n* = 3)
**Orbit area**	(707.2)	593.9 (*n* = 2)	563.5 (*n* = 3)	892.5 (*n* = 2)	727.3 (*n* = 5)	763.0 (*n* = 3)	791.2 (*n* = 3)	630.0 (*n* = 3)	718.0 (*n* = 8)	611.8 (*n* = 7)	782.3 (*n* = 7)	678.6 (*n* = 4)	847.9 (*n* = 6)	702.9 (*n* = 3)
**Malar height**	34.2	31.3 (*n* = 3)	25.6 (*n* = 4)	26.1 (*n* = 3)	25.0 (*n* = 4)	33 (*n* = 1)	31.8 (*n* = 14)	24.6 (*n* = 9)	27.6 (*n* = 7)	23.6 (*n* = 7)	34.9 (*n* = 10)	29.5 (*n* = 12)	43.6 (*n* = 5)	32.7 (*n* = 3)
**Relative malar height**	(1.3)	1.4 (*n* = 2)	1.2 (*n* = 3)	0.9 (*n* = 3)	1.0 (*n* = 3)	1.2 (*n* = 1)	1.3 (*n* = 14)	1.1 (*n* = 9)	1.1 (*n* = 7)	1.0 (*n* = 7)	1.4 (*n* = 10)	1.2 (*n* = 12)	1.7 (*n* = 5)	1.3 (*n* = 3)

**Notes:** Orbit height defined as the maximum distance between the inferior and superior orbit borders. Orbit width defined as the maximum distance between the lateral and medial orbit borders. Orbit area is defined as orbit width x orbit height and is measured in mm^2^. Malar height defined as the distance between orbitale inferior/zygoorbitale and zygomaxillare inferior. Relative malar height defined as malar height/orbit height. *P*. *angusticeps* specimens include KA 194, KA 195, CO 100, CO 135A/B, CO 101, GV 4040, and HGD 1249. *P*. *izodi* specimens include TP 7, TP 12, SAM 11728, T10, T13, UCMP 125854, UCMP 125855, UCMP 125856, STS 262, T89-11-1, and SWP UN-2. *P*. *robinsoni* specimens include UCMP 56797, UCMP 56786, and M3147. No measurements were available for *P*. *robinsoni* males. Numbers in parentheses represent estimates. Values for each taxon represent averages. See also Fig 4 and Table A in S1 Dataset.

**Fig 5:**
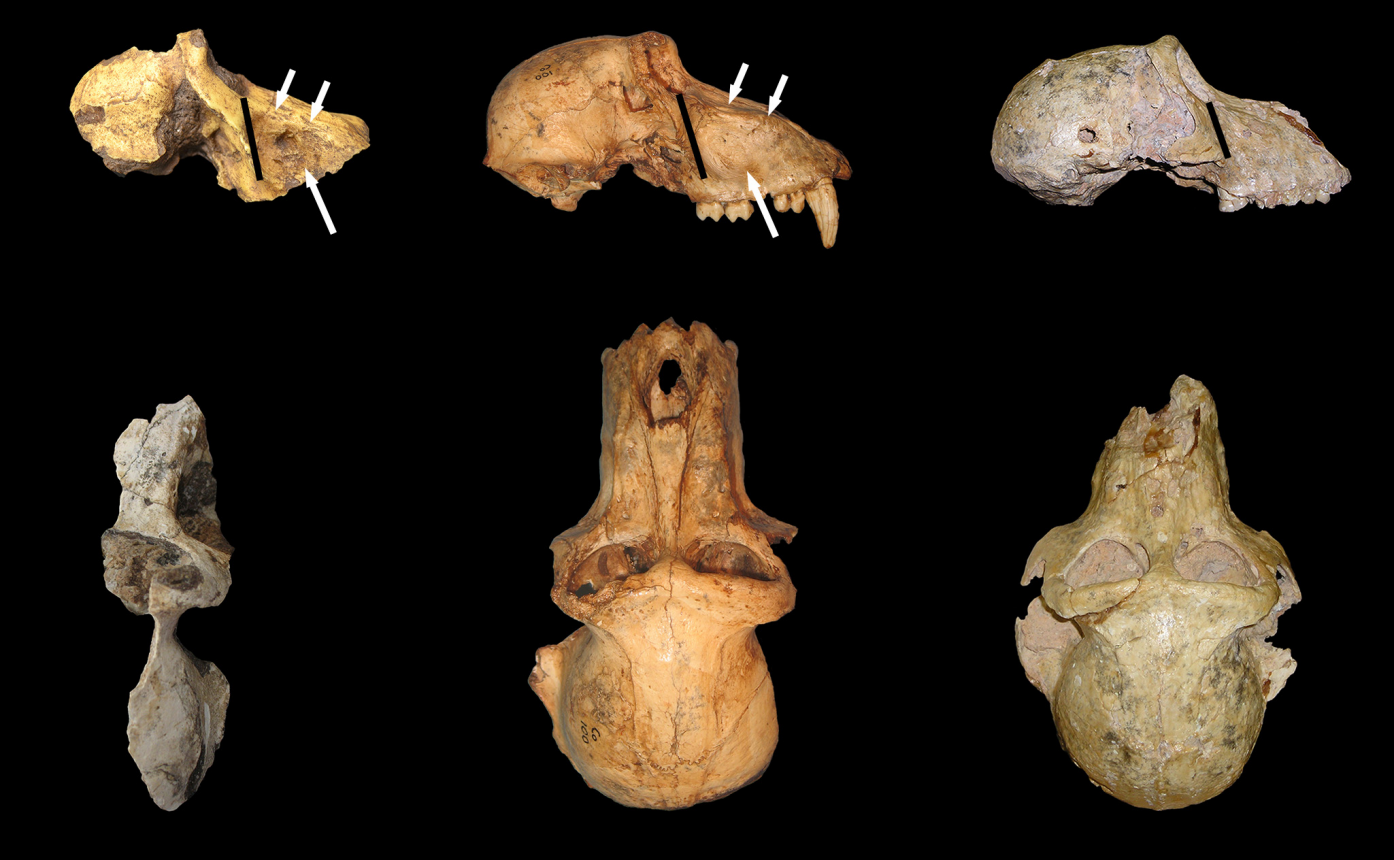
Comparison of morphology in UW 88–886 (left), *P*. *angusticeps* males (CO 100, center), and *P*. *izodi* males (TP 89-11-1, right). Top: Lateral view, specimens scaled to approximately the same cranial height. Note the tall malar region (black bar), prominent maxillary ridges and deep maxillary fossae (white arrows) in UW 88–886 and *P*. *angusticeps* compared with *P*. *izodi*. Bottom: Dorsal view, specimens scaled to approximately the same cranial width. Note the longer, narrower muzzle in *P*. *angusticeps* compared to *P*. *izodi*, and again the prominent maxillary ridges and deep maxillary fossae in UW 88–886 and *P*. *angusticeps* compared with *P*. *izodi*.

The zygomatic bone bulges outward slightly lateral to a series of infraorbital/maxillary foramina, then pinches inferiorly, forming sharp cheekbones bordering the pronounced infraorbital/maxillary fossa. The right lateral border of the orbit is intact from the frontal-zygomatic suture to the beginning of the temporal process of the zygomatic. The zygomatic arch is not preserved (Figs 2 and 3). Malar height, as characterized by the minimum distance from inferior orbital margin to the inferior border of zygomatic process of the maxilla, is relatively tall in UW 88–886, a feature it shares with other *P*. *angusticeps* specimens and similarly-sized modern *P*. *hamadrayas* baboons (e.g., *P*. *h*. *cynocephalus*) to the exclusion of *P*. *izodi* (Tables 1–3; Figs 4 and 5). On the lateral orbit border, there are two zygomatico-facial foramina, the larger in the central body of the zygoma and the smaller is placed superiorly near the frontal-zygomatic suture. The zygomatic root begins above the maxillary alveoli at the level of the M^2^–M^3^ contact. The lateral margin of the zygomatic appears to flare slightly outward (laterally).

The preserved portion of the muzzle dorsum is flat to slightly rounded moving medially, and there is strong development of a prominent maxillary ridge bordering the lateral aspect of the muzzle dorsum. The superior rim of the nasal aperture is broken in this specimen; however, what is preserved of the superior portion of the rim appears to have been rounded. The premaxillae do not extend up along the nasals as far as in some other specimens of *P*. *angusticeps* (e.g., female CO 101). There is a slight groove medial to the canine juga extending to a large infraorbital foramen. Lateral and inferior to this foramen, there are several smaller maxillary foramina. The lateral portion of the muzzle is dominated by a deep and pronounced maxillary fossa extending into the zygomatic and maxillary bone inferior to the orbital margin (i.e., the infraorbital plate).

In dorsal view, there is evidence for a strong supraorbital region based on the preserved morphology of the right superolateral orbit border. The superolateral portion of the orbital rim is relatively flat and the supraorbital torus has a sharp border demarcated posteriorly by the temporal line with a distinct lip above the post-orbitally constricted frontal bone. There is marked post-orbital constriction with evidence of a probable post-orbital sulcus, not as much constriction as in *Theropithecus*, but a similar degree to that observed in typical male *P*. *angusticeps* and *Papio hamadryas* specimens (Figs 2–5). Also similar to *P*. *angusticeps* and modern *Papio hamadryas* males, in particular, UW 88–886 clearly displays pinched temporal lines as far medially as they are preserved, although it is unclear whether they would have met posteriorly to form a slight sagittal crest. The temporal lines emanate from the supero-lateral corner above the orbits medial to the post-orbital constriction of the frontals.

## Discussion

Based on preserved morphology, UW 88–886, a presumed male, is most similar to the South African taxon *Papio angusticeps* among known Plio-Pleistocene baboon populations. The combination of an anteorbital drop, long and narrow muzzle, well-developed maxillary ridges, well-developed and deep maxillary fossae, and a relatively tall malar region make the specimen statistically distinct from other Plio-Pleistocene *Papio* species and most similar to *P*. *angusticeps* as well as modern *P*. *hamadryas* subspecies (Tables 1–3; Figs 4 and 5). The identification of *P*. *angusticeps* at Malapa has broader implications for baboon evolution and hominin biochronology as well.

Modern baboons (extant *P*. *hamadryas* ssp.) represent one of the most successful living primate radiations, with populations distributed all across sub-Saharan Africa and into the Arabian Peninsula. Despite their evolutionary success, modern baboon origins in the fossil record are not well understood or agreed upon. In East Africa prior to the Middle Pleistocene, there are craniodental fragments from various localities that possibly represent the genus *Papio*, but none of these fossils are definitively diagnostic (e.g., [20–25]). Fossils more clearly attributable to the modern *Papio hamadryas* population appear in the Middle Pleistocene at Olduvai Gorge, Asbole, and Bodo [26–29].

During the South African Plio-Pleistocene, at least two species of “small-bodied” papionins exist that have been typically placed into the genus *Papio*: *P*. *izodi* and *P*. *angusticeps*. *P*. *izodi* is currently recognized at Taung as well as Members 2 and 4 at Sterkfontein [30–33], suggesting an age range anywhere from ~3.7–2.0 Ma depending on the estimate (e.g., see [30, 34] for Taung; see [34–35] for most recent estimates of Members 2 and 4 at Sterkfontein). *P*. *angusticeps* on the other hand, is securely documented at sites generally suggested to be less than ~2.0 Ma, such as Kromdraai A and Cooper’s A. One exception might be the *P*. *angusticeps* population at Haasgat, which was recently argued to be slightly older, between ~2.58–1.95 Ma, with a more likely range for most fossils between ~2.3–2.0 Ma [36–37]. The age of *P*. *angusticeps* at Gladysvale is difficult to assess at this time as much of the faunal material was initially collected from ex situ breccia blocks of unknown provenience [34].

While some authors lump both *P*. *izodi* and *P*. *angusticeps* into a single taxon (*P*. *izodi* sensu lato; e.g., [22, 38]), other experts recognize them as different taxa (e.g., [30–31, 39]). Some authors even suggest that *P*. *angusticeps* is most likely a subspecies of the modern baboon radiation (i.e., *P*. *hamadryas angusticeps*) and note many similarities with modern *P*. *h*. *kindae* and *P*. *h*. *cynocephalus* populations, in particular [30, 33, 40]. Similar to East Africa, specimens definitively attributable to *P*. *hamadryas* appear in southern Africa by the Middle Pleistocene, best represented by the distinct subspecies *P*. *h*. *botswanae* in Botswana [41] and the South African Pleistocene/Holocene *P*. “*spelaeus*” specimen of unknown provenience that appears to represent a large *P*. *h*. *ursinus* male [22, 41–42].

*P*. *angusticeps* shares features with modern *P*. *hamadryas* such as a relatively tall malar region, relatively small orbits, a definitive anteorbital drop, deep maxillary/suborbital fossae, pronounced maxillary ridges in males, a definitive anteorbital drop, a relatively narrow muzzle, a relatively long muzzle, and relatively small teeth (Table 1; Fig 5). When Freedman [42] transferred the species to *Papio* from *Parapapio* (originally *Parapapio angusticeps* Broom, 1940), he also noted that *P*. *angusticeps* cranial morphology closely resembles that of *P*. *h*. *ursinus*, except that the maxillary ridges are slightly weaker and the maxillary fossae are slightly deeper in *P*. *angusticeps*. *P*. *izodi*, on the other hand, is much more primitive in that it displays a relatively short malar region, relatively large orbits, a variable anteorbital drop, shallower maxillary/suborbital fossae (particularly in males), little to no development of the maxillary ridges in males, a relatively broad muzzle, a relatively short muzzle, and relatively large teeth (Tables 1 and 3; Fig 5; see also [30–31, 33]). In fact, one recent phylogenetic analysis places *P*. *izodi* outside of the crown African papionins and questions its status as a member of the modern genus *Papio* at all [33]. In any case, it is clear that the morphology of *P*. *izodi* is distinct from that of *P*. *angusticeps* as well as the modern baboon populations, *P*. *hamadryas* ssp. (Tables 1–3; Figs 4 and 5).

Excluding *P*. *izodi*, there are a few additional South African fossil *Papio* specimens that are relevant to the origins of the modern baboon. *Papio robinsoni* is a large form of *Papio* (larger on average than *P*. *izodi*, *P*. *angusticeps*, and most modern subspecies) securely documented at sites estimated to be anywhere between ~2.6–1.5 Ma: Swartkrans Member 1, Skurweburg, Bolt’s Farm Pit 23, and Drimolen; it is also less securely identified at Sterkfontein Member 4 [30, 42–46]. While *P*. *robinsoni* has also been argued to be a member of the modern *Papio hamadryas* radiation by some (e.g., [30, 33, 38, 44]), there are possible reasons to suspect it is distinct. *P*. *robinsoni* clearly displays synapomorphies with modern *P*. *hamadryas* such as a distinct anteorbital drop, large overall size, definitive maxillary ridges, a relatively long and narrow muzzle, relatively small orbits, relatively small teeth, and definitive maxillary fossae in males (Tables 1 and 3). However, there are also consistent differences between *P*. *robinsoni* and modern *P*. *hamadryas* ssp. *P*. *robinsoni* displays weaker development of the maxillary and mandibular corpus fossae than modern baboons (Table 1), maxillae that occasionally meet in the midline (covering the nasals near nasion), and nasals that lie below the level of the maxillary ridges on the muzzle dorsum [40, 42]. Our interpretation is that these features are primitive relative to modern baboons, particularly the slight development of the facial fossae. *P*. *angusticeps*, on the other hand, displays no features that are obviously distinct from the modern *P*. *hamadryas* populations (see Tables 1 and 3), suggesting that it is possibly the first well-documented modern *P*. *hamadryas* subspecies in the fossil record (i.e., *P*. *h*. *angusticeps*). While *P*. *angusticeps* displays slightly deeper fossae and less-developed maxillary ridges on average when compared to *P*. *h*. *ursinus* [42], similar morphology to *P*. *angusticeps* can be found among the other *P*. *hamadryas* susbspecies. A more complete and formal taxonomic reassessment of *P*. *angusticeps* and *P*. *robinsoni* is beyond the scope of this paper, but future studies may further support the sinking of *P*. *angusticeps* into *P*. *hamadrayas* (as originally suggested by Delson [30]), depending on the species concept being used. In particular, given the quantitative morphometric differences documented among modern *P*. *hamadryas* populations [41, 47], future 3D morphometric studies including both *P*. *angusticeps* and *P*. *robinsoni* may help illuminate the affinities of these taxa relative to modern *P*. *hamadryas* populations and relative to the recently described *P*. *h*. *botswanae*.

Interestingly, recent molecular dates place the origin of the modern *P*. *hamadryas* radiation between ~1.8 and 2.2 Ma in South Africa [48–51], which closely brackets the estimated age of the Malapa specimen at ~2.026–2.36 Ma. Thus, the *P*. *angusticeps* specimen described here may not only represent the earliest appearance of *P*. *angusticeps*, but potentially the earliest appearance of the modern baboon population in the fossil record, in almost perfect agreement with molecular estimates. While it is true that the molecular clock must always be calibrated through the fossil record and, therefore, there is always a bit of circular reasoning involved, it is still noteworthy and perhaps reassuring whenever new fossil discoveries seem to confirm molecular estimates, thereby lending support to hypothesized divergence dates. Because the estimated age of the Malapa fauna seems well-constrained around the dated ~2.026 Ma flowstone (see Geological Context above), the presence of *P*. *angusticeps* also assists in the biochronological assessment of other sites where *P*. *angusticeps* is documented, including Gladysvale, Haasgat, Kromdraai A, and Cooper's A [30, 33, 42, 44, 52–53]. In particular, the Malapa specimen supports recent revised estimates for the age of the Haasgat fauna (and *P*. *angusticeps*) between ~2.3–2.0 Ma, and suggests that Haasgat and Malapa together document a more secure first appearance date (FAD) for *P*. *angusticeps* between ~2.3–2.0 Ma. Cercopithecoid taxa such as *P*. *angusticeps* have long been used as important biochronological markers in age assessments of the South African Plio-Pleistocene hominin sites (e.g., [30–31, 44, 32, 54]), thus a more certain FAD for *P*. *angusticeps* may consequently lead to a slight adjustment in the estimated chronology of other hominin sites pending future faunal correlation analyses.

## Supporting Information

S1 Dataset Raw measurements used in this study (Table A). (XLS)
